# Library of Medicine: Vol.VI.—A System of Midwifery

**Published:** 1841-10

**Authors:** 


### Art. XII.

1. *Library of Medicine:* Vol. VI.—*A System of Midwifery.* By Edward Rigby, m.d., Physician to the General Lying-in-Hospital, Lecturer on Midwifery at St. Bartholomew's Hospital, &c. &c.—*London,* 1841. 8vo, pp. 314.
2. *The Principles and Practice of Obstetric Medicine and Surgery, in reference to the Process of Parturition; with One Hundred Illustrations on Steel and Wood.* By Francis H. Ramsbotham, m.d., Consulting Physician in Obstetric cases to and Lecturer on Obstetric and Forensic Medicine at the London Hospital.—*London,* 1841. 8vo, pp. 672.
3. *The Elements of Obstetric Medicine, with the Description and Treatment of some of the principal Diseases of Children.* By David D. Davis, m.d. m.r.s.l., Professor of Obstetric Medicine in University College, and one of the Physicians to University College Hospital. Second Edition.—*London,* 1841. pp. 1002.

The increased and increasing degree of attention that is devoted to the department of Midwifery upon the continent of Europe is amply shown in the great number of systematic treatises upon that subject that have of late years appeared in France and Germany. In the French language we have elaborate and excellent general works on this branch of medical science published within the last fifteen or twenty years, by Gardien, Capuron, Maygrier, Boivin, Velpeau, Halmagrand, and Moreau, besides several smaller manuals, by Duges, Hatin, Chevreuil, and others. Almost every German medical school has of late sent forth a text-book of its own on the subject of obstetric medicine. We need only refer, amongst others, to the learned systematic works and hand-books of Busch, Carus, Kilian, Horn, Joerg, Siebold, Froriep, Wilde, &c. The modern medical literature of England contains many valuable isolated essays and monographs on particular departments of midwifery, but comparatively few general synopses of the principles and practice of this branch of the profession. We point out, however, with pleasure, as exceptions to this dearth of systematic compendia of obstetric science, the well-known and esteemed work of Dr. Burns, the clear and practical Synopsis of Dr. Merriman, the short Manuals of Drs. Conquest, Reid, and Maunsell, the excellent Introduction to Midwifery of Dr. Campbell, (a work highly valuable from its presenting the best account now extant of the late Dr. Hamilton's doctrines and practice,) and the notes of the lectures of Blundell and Gooch, that have been published by some of their pupils. It gives very great pleasure to see added to this list, and that within the current year, the three works at the head of the present article. The simultaneous publication of systematic works on midwifery, by three of the most distinguished obstetric teachers in the metropolis, is in itself a sufficient sign of the greater importance that has become attached to the study of obstetric medicine in our own country; and we willingly embrace the opportunity thus afforded us of directing the attention of our practical readers to some of the principal changes and improvements in doctrine and treatment which the researches of recent years have effected in scientific midwifery.

In some of the preceding volumes of our Journal we have already taken occasion to discuss at some length the subjects of embryology and the physiology of generation. We set these subjects aside therefore at present, both for this reason and because we must not search in the works of such practical men as Drs. Davis, Rigby, and Ramsbotham, for what is new in this wide and complicated field of investigation. In the following remarks we shall confine ourselves rather to such notices as our space will permit of the opinions of these gentlemen in natural, morbid, and instrumental midwifery. We shall speak more particularly of the works of Drs. Rigby and Ramsbotham, as we have already in our Third Volume treated at some length of Dr. Davis's Elements of Obstetric Medicine, as formerly published in the more extended quarto form.

The different authors whose works are now before us classify differently the forms and varieties of labour, normal and pathological. It is a matter of little moment which of their arrangements we follow in considering and contrasting their particular opinions. We shall adopt, therefore, that of Dr. Ramsbotham (the old classification of Denman), both because we consider it as good as any that has hitherto been proposed, and because it is the one with which we believe our readers are already most intimate.

In looking to the subject of natural labour, the first important topic which meets us is, that of the mechanism of the process. The mechanism of parturition, or the mode in which the fœtal head is applied to and enters the pelvis of the mother, and the different changes which it undergoes in its relations during its passage from the brim through the cavity and outlet, independent of its physiological and anatomical interest, is undoubtedly of the highest importance in its bearings on practical midwifery. It forms indeed the very basis both of the theory and of the art. Its mere mechanical nature may, in the eyes of some, place it inferior in medical interest to other pathological and physiological questions in the department of obstetric medicine, but it is certainly subordinate to none in actual utility.

We cannot, with Dr. Rigby, see how any one can detect, with certainty, the deviations that are apt to occur from the usual course of labour, unless he be thoroughly acquainted with every step of the mechanism in the natural process. Nor can we see how any one can expect to assist instrumentally with perfect certainty, and to the fullest advantage, the various evolutions and motions of the fœtal head in the different varieties of labour, without knowing what the most natural and consequently the most easy evolutions of each particular variety actually are. No remark, however, is certainly more true than that made by Dr. Denman, that natural labour was the last sort of labour properly studied and properly understood, and we have no hesitation in pronouncing our opinion, even at the present day, that though it is a subject worthy of the greatest attention it has, at least in this country, hitherto excited very little notice, and though a topic on which, above all others, it is necessary to have correct opinions, there are certainly few departments in midwifery upon which the practitioners of Great Britain entertain, generally speaking, more loose and more incorrect ideas.

It is curious in looking back to the past history of practical midwifery, to weigh the talent and ingenuity that have been bestowed upon the fabrication of instruments to assist, as it was called, in difficult labours, and at the same time to reflect that the fundamental facts on which the principles of their application and employment rest, were entirely unknown to their inventors. Thus, when the Chamberlens first invented the forceps to drag the fœtal head through the pelvis, they neither knew the manner in which the fœtal head enters the pelvis, nor any one of the relations which it occupies in its passage through the pelvic cavity. They and many of their followers endeavoured to assist nature in her movements before they knew what these movements were.

In making these remarks we beg to state that we are perfectly aware that various authors, both British and foreign, have long ago endeavoured to describe more or less minutely the mechanism of natural labour and its different modifications. Their descriptions, however, were drawn from theory rather than from experience, from fancy more than fact, from rude experiments made with the dry bones of the head and mother's pelvis, and not by careful and minute observation of the process as it is conducted in nature. Hence the accounts of different authors, as might readily be expected, show the greatest discrepancy and discordance even on the same points. Whole paragraphs, and whole pages, as Naegele in his comments upon this subject has remarked, are to be found in obstetric works, with their contents respecting the mechanism of parturition perfectly disagreeing with one another, and still more disagreeing with nature. Relations and movements are described which do not occur, and those that do occur are in many instances left undescribed. Authors have but too often shown how they would move the head through the pelvis, if they had the direction of it, rather than endeavoured to show how nature herself conducts the process.

In an article such as the present it is impossible for us to enter at length into the various questions connected with the mechanism of labour. We will offer merely a few remarks to illustrate some more general facts with regard to it, and to show the truth of what we have previously stated in regard to the discrepancy of opinions that long prevailed upon the subject.

In the first place, it is almost unnecessary to observe that all admit that the head of the child presents at the brim of the pelvis in a very large proportion of cases of labour. This proportion is as great as 94 or 95 in the 100; or, in other words, the exceptions to this general rule are only about 1 in every 30 cases of labour according to Collins, 1 in 33 according to Osiander, 1 in 34 according to Carus, or as Meckel has derived it from various obstetric statistics, only 1 in 35.

But the next question in the consideration of the mechanism of parturition is a much more undecided one, namely, in what direction does the head of the child enter the brim of the mother's pelvis? Important as an accurate knowledge of this fundamental fact is to an acquaintance with the subsequent evolutions which the child's head undergoes, and to the practical management of labour, it was not till the middle of the last century that the slightest approach was made to an accurate opinion on this subject. Since that period several different doctrines have been promulgated in respect to the direction in which the long or antero-posterior diameter of the child's head enters the brim at the commencement of the process of parturition.

A glance at the history of these opinions will illustrate the subject better than any formal comments on it.

1. By all the older authors the head seems to have been supposed to enter the superior margin of the pelvis, in the same relation in which they observed it to emerge from the outlet, namely, with the sagittal suture or long diameter parallel to the conjugate or antero-posterior diameter of the brim.

2. In 1742, Sir Fielding Ould, of Dublin, called this first opinion in question, and maintained that, in entering the pelvis, the long diameter of the head lay parallel to the transverse diameter of the brim. We shall quote his own words, as his work is now somewhat rare, and his original observations on this point little known:

> "When a child presents itself naturally, it comes with the head foremost, and (according to all authors that I have seen) with its face towards the sacrum of the mother, so that when she lies on her back, it seems to creep into the world on its hands and feet. But here I must differ from this description in one point, which at first sight may probably seem very trivial; the breast of the child does certainly lie on the sacrum of the mother, but the face does not, for it always (when naturally presented) is turned either to the one side or the other, so as to have the chin directly on one of the shoulders." (Ould's Midwifery, p. 28.)

From this it is evident that Ould believed the child's head to be so greatly twisted round on its trunk "as to have the chin on one shoulder." In 1752, Smellie corrected this notion with regard to the contortion of the child's neck, but otherwise fully adopted Ould's views of the direction of the head. In 1771 Deleurye first promulgated the same opinions in regard to the position of the head in labour in France; and in later times Schmitt and Mampe, in Germany, have upheld the same doctrine.

3. In 1771 two celebrated obstetric essays were published upon the continent, the one by Saxtorph of Copenhagen, and the other by Solayres de Renhac of Montpellier, containing observations made independently of each other, but both agreeing in this one great fact—that the long diameter of the head of the child in natural labour entered the pelvis in a direction neither parallel to the conjugate nor to the transverse diameter of the brim, but parallel to one of its *oblique* diameters; that is, with the sagittal suture running in a line directed at one extremity to the sacro-iliac synchondrosis behind, and to the foramen ovale anteriorly. They further both showed of the two oblique diameters, the long axis of the head, in a very large proportion, occupied the right, or that running between the right sacro-iliac synchondrosis and left foramen ovale.

Baudelocque, the pupil of Solayres, adopted the opinions of his master as one of the great foundations of his classification of labours, though he did not adhere exclusively to his master's views. The great influence of Baudelocque over the French and other schools of midwifery has spread widely the knowledge of this doctrine of the oblique position of the head at the brim. It was assuredly, however, not until Professor Naegele of Heidelberg published his incomparable essay on the Mechanism of Parturition in 1818, that the great and almost exclusive predominance of the oblique direction of the head at the commencement of labour was fully acknowledged. His opinions have daily gained more and more ground on the continent of Europe, but though his essay was translated into our own language in 1828, we regret to add that the older opinions on the subject of parturition are still taught in many of our British schools. We feel certain, however, that the more his essay is studied, and the more his observations are accurately compared with what occurs in nature, the more will Naegele's opinions be found to gain weight with the practitioners of this country.

From the preceding slight historical sketch it is evident that it has been supposed that the head of the child may, at the commencement of labour, enter the brim of the pelvis in four different positions; namely, with its long diameter, 1st, parallel to the conjugate diameter of the pelvis; or, 2d, parallel to its transverse diameter; or, 3d, parallel to its right oblique diameter; or, 4th, to its left oblique diameter. The first two have been sometimes named the direct or straight positions of the head, the last two the oblique or diagonal. Further, the long diameter of the head may be placed in each one of these pelvic diameters, in two different relations, viz., with the forehead directed to one extremity of it, or the head being completely reversed, the occiput may be directed to the same point.

We make these remarks in order to show the foundation on which different authors have based the classifications of cranial presentations that they have proposed. Various writers have described the head as capable of entering the brim of the pelvis in natural labour in all of these eight positions; others have reduced them to six, and among the authors who have done so, there are some who, as Baudelocque and his followers, have thrown out the two positions referrible to the conjugate diameter of the brim, and others who, as Lachapelle, on the contrary, have renounced the two positions referrible to the transverse diameter. All our best observers for the last sixty or seventy years have reckoned the oblique positions as the most common. Capuron, Naegele, &c. consider the four oblique as the only positions seen in cases of natural labour, and Dr. Rigby has endeavoured still further to refine upon this, by reducing the natural presentations of the head at the brim to the two that can occur in the right oblique diameter alone, namely, 1st, with the face pointed towards the right sacro-iliac synchondrosis, and the occiput to the left acetabulum; and 2dly, with the face looking to the left acetabulum, and the occiput to the right sacro-iliac synchondrosis.

The different authors who have adopted the different classifications of cranial presentations to which we have referred have affixed to each an ordinal number to designate them, but, unfortunately, they have but too often employed the same number to designate different presentations. Hence we find, for example, the third or fourth positions of one author not at all corresponding to the third or fourth of others. It is unnecessary to comment on the discordance to which this has led. In taking up each separate author on the subject, we have first to teach ourselves his particular language on this point, before we can either understand his observations or the rules of treatment which he lays down. As a key to the obstetric reader in his study of some of the more valuable authors of the present day, we beg to offer the following table:

**Table, showing the Numerical Order of Classification followed by different Authorities in their systematic Arrangements of Cranial Presentations.**

| Numbers affixed to presentations, by | | | | | | Description of Presentation. |
|---|---|---|---|---|---|---|
| Rigby. | Naegele. Capuron. Maygrier. Duges. Halmagrand. | Baudelocque. Dubois. Gardien. Davis. Dewees. | Lachapelle. | Boivin. Flamant. Moreau. | Ramsbotham. | Anterior part of Cranium pointing |
| 1 | 1 | 1 | 1 | 1 | 3 | to Right Sacro-iliac Synchondrosis. |
| | 2 | 2 | 2 | 2 | 4 | ,, Left ,, ,, |
| 2 | 3 | 4 | 3 | 4 | 6 | ,, Left Foramen Ovale. |
| | 4 | 5 | 4 | 5 | 5 | ,, Right ,, |
| | | 3 | | 3 | 7 | ,, Promontary of Sacrum. |
| | | 6 | | 6 | 8 | ,, Symphysis Pubis. |
| | | | 5 | 7 | 1 | ,, Right Os Ilium. |
| | | | 6 | 8 | 2 | ,, Left ,, |

The classification of head presentations adopted by the authors whose works we have under review will be sufficiently apparent from this table. In it we have placed Dr. Davis as following Baudelocque's arrangement, as he evidently professes to do at p. 621 of his work, though at the same time we must remark that, after announcing it, he himself does not strictly adhere to that arrangement, for (and we could quote other examples) while at p. 622 he describes the third position as that in which the occiput lies behind the symphysis pubis, in p. 808 he describes the same third position as that in which the right ear lies immediately behind the symphysis pubis and the occiput directed to the left side of the pelvis. We do not stop to comment on the confusion to which such a discrepancy of statement must lead in the mind of the obstetric student.

Though Dr. Rigby has admirably described the mechanism of parturition in his work, we confess that we feel somewhat dissatisfied with his innovations upon the arrangement and nomenclature of Naegele, whose work he himself first introduced to the British public.

We are so old as to dislike to learn a new language to designate objects with which we have long been familiar. In other respects we can earnestly recommend Dr. Rigby's chapter on the mechanism of labour to the attention of all those who wish to obtain accurate ideas with regard to this matter.

Dr. Ramsbotham has, as will appear from the foregoing table, proposed an arrangement of cranial positions with a numerical order peculiar to himself. We have the same objection, of unnecessary innovation, to urge against him in this respect as we have just made against Dr. Rigby. Further, we certainly consider the number of presentations that Dr. Ramsbotham has described as too great and complicated. We are not inclined with some authors absolutely to deny that the direct position of the head in the conjugate and transverse diameters cannot possibly occur. We could, we think, if this were a proper place, point out some particular elongated forms of the brim of the pelvis, which would almost oblige nature, if we may so speak, to enter the child's head in one or other of these diameters. But these forms are so exceedingly rare, and these varieties of presentation so very seldom occur in practice, when the child's head and mother's pelvis are of the natural size, that they may certainly be kept out of any classification of labours that are purely normal. Obstetric writers have almost always numbered the presentations in correspondence with the comparative frequency according to which they respectively occur, or, as Naegele has done in regard to his second position, in deference to the numbers already appended to them by others. Dr. Ramsbotham has set aside both these circumstances as the basis for his nomenclature. The numbers he has appended to the positions are, we believe, different from those appended to them by any preceding author; and though he inadvertently states, at p. 135, that his first four presentations are "by far the most frequent," yet his fourth (the second position of Naegele) is certainly seldom met with, and is greatly less frequent than his sixth (the third position of Naegele), while both his first and second are alleged by Baudelocque, who introduced them into his own classification of presentations, to be so exceedingly uncommon, that he had himself never met with one instance of it out of many thousand cases; Boivin found them twice in 19,000 labours, and they are hence entirely rejected from the works of most of our recent and best authors.

After this last remark we could have wished that our space had permitted us to show, by the statistical reports of Lachapelle, Boivin, Naegele, Duges, Halmagrand, and others, the comparative proportion of instances in which the different positions of the head have been ascertained to occur in actual practice. It is an important topic, and one to which we may recall the notice of our readers at an early period. In the meantime we shall content ourselves with presenting the few following calculations of the per centages of frequency of the four oblique positions of the head.

The *first* position of Naegele (with the child's face to the right sacroiliac synchondrosis) has occurred to that author in his own practice in 69 per cent. of all his head presentations, to Halmagrand in 74 per cent., to Madame Lachapelle in 77 per cent., and to Madame Boivin in the ratio of 80 per cent.

The *fourth* position of Naegele (with the child's face directed to the right foramen ovale) has occurred in his own practice in the very small proportion of ·03 per cent. It has been met with equally rarely by others. Lachapelle and Halmagrand have found it in ·04 per cent., and Boivin in ·05 per cent.

So far we find a striking correspondence in the different observations of these authors, in a series of cases amounting in all to nearly 60,000. Their results, however, are more discrepant as regards the relative frequency of the second and third positions of Naegele. They all nearly agree, be it remarked, in regard to the absolute proportion of cases in which the face is directed to the left side of the pelvis, in the same way as they all nearly agree as to the absolute proportion of cases in which the face is directed to the right side. They differ, however, much as to the proportionate number of instances in which the face being directed to the left side, is pointed originally towards the left sacro-iliac synchondrosis (constituting the *second* position of Naegele), or towards the left foramen ovale (constituting the same author's *third* position of the head). Thus while Naegele states that in above 1,290 cases he only met with the *second* position in one instance, or in the proportion of about ·07 per cent., Halmagrand describes it as occurring in 5 per cent. of his cases, Boivin in 19 per cent., and Lachapelle in as great a ratio as 21 per cent. On the other hand, while Naegele found not less than 359 cases of the *third* position in 1,210 cases of cranial presentations, or as many as 29 per cent. of instances in which the face looked at the commencement of labour to the left groin, Lachapelle reports only ·077, and Boivin 0·5 per cent. as presenting in the same situation.

Various explanations have been attempted of these and other reported differences of results in the comparative frequency of the second and third positions. The observations of Naegele were, we believe, made by himself in most if not in all the cases to which he refers, most of those of Lachapelle and Boivin by females attached to the Maternité Hospital of Paris. The observations at the hospital were collected, without there having been previously strongly pointed out a great source of fallacy in confounding the second and third positions, and those of Naegele were conducted with a perfect knowledge of and a view to this fallacy. We are inclined, therefore, both from what we have ascertained by our own experience, and from what we know of the high character and candour of Naegele, to attach more credit to the results of his observations than to those who have been opposed to him on this point. And we further believe that the great source of fallacy to which we have alluded and which is so apt to deceive is this, that if the face present to the left, and the position of the head be not examined till it is emerging through the outlet, it will in a very great proportion of cases be found by that time turned round into the second position, though it originally presented in the third. We shall afterwards see that this rotation of the face backwards is the general and normal course followed by cases of the third position.

We have not space to pause and point out the anatomical reasons which, on physical principles, prevent the occurrence of the *direct* positions, and lead, on the other hand, to such an overwhelming proportion of cases in the right oblique diameter. The subject, however, is one of great interest.

The part of the child's head that lies lowest in the pelvis, or in other words, the exact nature of the presenting part in cranial presentations, has given rise to almost as great difference of opinion as the direction of the head itself. Daventer long ago pointed out the oblique position of the fœtus and the uterus itself in relation to the vaginal and pelvic cavity; but, notwithstanding his observations, we find misstatements in works up even to the present time in respect to this point, and more especially in respect to its influence upon the regulation of the presenting part. The anterior fontanelle, sagittal suture, and still more commonly the vertex, have each been alleged to be the parts that the finger of the accoucheur first touches upon. The acknowledged obliquity of the child's body and head in relation to the vaginal canal might have been sufficient alone to have taught the fact, that it could not be a *mesial* part of the child's head that presented in natural labour, and we believe that every one now, who has actually studied the matter in nature, agrees that what Naegele first distinctly pointed out, that the posterior and superior portion of one or other of the parietal bones is first felt on examination by the accoucheur. When the head presents with the face looking towards the right side of the pelvis, it is the right parietal bone that first descends; when the face looks towards the left, it is the left parietal bone. On the above points Dr. Rigby offers the following sound generalizations, in his consideration of the mechanism of head-cases of the first position, or with the face looking to the right sacro-iliac synchondrosis:

"The head enters, passes through, and emerges from, the pelvis obliquely; and this is the case not only as to its transverse diameter, but also as to the axis of its brim; the side of the head being always lowest or deepest in the pelvis. This shows the beautiful mechanism of the process, for on account of its oblique position, there is no moment during the whole labour at which the greatest breadth (still less length) of the head is occupying any of the pelvic diameters; even at the last, when the head is passing under the pubic arch, the complete obliquity of its position, in order that it should take up the least possible room, is very remarkable, for the ring of soft parts, by which the head is now encircled, passes obliquely across it, running close behind the left and before the right parietal protuberance. The head never advances with the occiput, forwards, under the pubic arch, as is stated in works on midwifery, still less with the sagittal suture parallel to the antero-posterior diameter of the pelvis; for the direction of the right lambdoidal suture, as also of the posterior fontanelle, and the position of the cranial swelling, or caput succedaneum, as it has been called, completely prove the inaccuracy of such a theory, the sagittal suture crosses the left labium at an acute angle, the right lambdoidal suture being parallel with the left descending ramus of the ischium." (p. 126.)

He adds another important remark:

"Not less incorrect is the theory (for we can call it nothing else) of the head presenting with the vertex, and turning with its long diameter from the oblique into the antero posterior or conjugate diameter, and the face into the hollow of the sacrum, for it is disproved by all the above-mentioned facts, which careful examination during labour puts us in possession of. When the head is born, the face looks backwards and to the right, viz. to the back part of the mother's right thigh, for the shoulders are by this time passing through the pelvis in its left oblique diameter, the right shoulder being forwards and to the right, and lowest in the pelvis; it is also that which is first expelled." (p. 127.)

Dr. Ramsbotham, in the beautiful plates which accompany his work, has given us eight figures illustrative of the modes in which the head of the child may be supposed to enter the brim of the pelvis in each of his eight positions. In these his artist has unfortunately represented throughout the fœtal cranium as entering the pelvis directly, and not in the oblique mode in which it does in nature. In plate 35 indeed, the fœtal heads are seen, by looking at the basis of the cranium, to be somewhat oblique, but the artist has shaded them so that they look obliquely backwards instead of forwards. In plate 39, as representing the second stage of labour, the face is turned far too directly into the hollow of the pelvis. We much admire, however, plate 41, representing the position of the child immediately after the head is expelled. Dr. Ramsbotham's own description of the usual course of natural labour is in most respects excellent, as are his directions for its treatment. On the management, indeed, both of natural and morbid labours, we have a precision and minuteness in them that must render his work a valuable practical compendium for the student and practitioner. Dr. Ramsbotham recommends the first two fingers of the *left* hand to be used in vaginal examinations in the first stage in those cases in which the os uteri is situated so high that it cannot be perfectly investigated by the fore-finger of the right hand. We believe this to be a very useful direction. The artist in representing this mode of examination (plate 42) has incorrectly represented the whole hand, with the exception of the thumb, as introduced into the orifice of the vaginal cavity.

In supporting the perineum, Dr. Ramsbotham justly states that it is not necessary that we should make powerful pressure, nor resist the child's exit by the employment of any exertion. Continuing, as it were, the resistance and curvature of the sacrum by our hand we are only to afford passive support; to allow the head covered by the perineum to be protruded against our hand rather than forcibly press our hand up against the head. We must recollect further that, as Dr. Rigby particularly mentions, "the passage of the head is not the only moment of danger to the perineum, for laceration is even still more liable to be produced during the expulsion of the shoulders; any slight rupture of the anterior edge is now apt to be converted into a considerable laceration, unless the support be continued until the thorax be expelled." As a specimen of the excellence of Dr. Ramsbotham's practical directions, and the vigorous style in which he lays down his rules and doctrines on this and other subjects, we extract the following short passage on an important point of treatment first very prominently brought under the attention of the profession by Mr. White of Manchester, viz. the treatment of the delivery of the body after the head is born :

"It used to be the custom to surround the neck with the thumbs and fingers of both hands, and forcibly extract the body the moment the head was in the world, for the purpose of liberating the woman from pain, and terminating the delivery as speedily as possible. Such practice is attended with double danger; great chance of injury to the child, by the tension of the neck, and no small probability of hazard to the mother, by the uterus being prematurely emptied. It is thus left in a flaccid state; the stimulus which previously disposed it to contract is suddenly taken away; that disposition ceases or is suspended; hemorrhage is induced; and necessity probably arises for the artificial removal of the placenta, and incalculable mischief is the consequence. Those persons who commend such meddling interference, and who estimate the skill of the obstetrical attendant by the rapidity with which he can extract the body after the head is born, found their eulogium on the most dangerous premises." (p. 173.)

Dr. Rigby describes with great minuteness and clearness the treatment, &c. of natural labour, and of the puerperal state. He directs the attention of his readers to auscultatory and other phenomena of labour that have hitherto been little described in English systematic treatises. We extract from his work some remarks relative to a source of prognosis of the duration of labour which we believe will be new to most of our readers:

"The celebrated Wigand of Hamburg considered that the form of the vagina would frequently furnish the means of a pretty certain prognosis as to the duration of labour; thus, if it were wide and yielding throughout its whole length, the labour would be quick, both at its beginning and termination; if on the other hand it were small, rigid, and contracted throughout, the labour might be expected to be of a very opposite character. If, on examination, the vagina is found roomy and well dilated at its upper part, but contracted and rigid near the os externum, the labour will be probably quick and easy during the first half, but slow and difficult afterwards; on the contrary, where the os externum is yielding and wide, but the upper portion of the vagina narrow, the labour may be expected to be slow at first, but to be brisk and active afterwards. We have already stated that the course of labour varies in every possible way; in some cases the same peculiar character of labour shows itself through two or three successive generations; hence it has been observed that very tedious or very violent and rapid labours sometimes seem to be hereditary; the mother, daughters, and grand-daughters being all remarkable for their lingering or rapid labours." (p. 111.)

We extract from Dr. Rigby's chapter on natural labour one more passage to show the different positions, &c. in which women are placed in different countries during the process of parturition. After speaking shortly of the history and form of the old labour-chairs of the continent, he remarks:

"In some remote parts of Ireland and also of Germany, the patient sits upon the knees of another person, and this office of substitute for a labour-chair is usually performed by her husband. Labour-chairs, as far as we are acquainted with their history, were never used in this country, nor have they been used for the last century in France, where the patients are usually delivered in the supine posture on a small bed upon the floor, which has not inaptly been termed *lit de misère*. A modification of the labour-chair is the labour-cushion first used by Unger, and afterwards by the late Professor Von Siebold of Berlin, and Professor Carus of Dresden; it is a species of mattress, with a hollow beneath the nates of the patient for receiving the discharges which take place during the labour. The patient is compelled to lie upon her back during the greater part of labour, and thus maintain the same posture for some time, which must necessarily become irksome and even painful to her. In this country and in Germany the patient is delivered upon a common bed, prepared for the purpose as above mentioned; in England she is placed upon her left side, the nates projecting to the edge of the bed, for the greater convenience of the accoucheur; in Germany, except in Vienna and Heidelberg, where the English midwifery has in a great measure been introduced by Boer and Naegele, the patient is delivered upon her back. In former times the supine posture was also used in this country, but for about a century the position on the left side has been preferred." (p. 109.)

The position upon the left side is still we have reason to know changed by some old practitioners at the present day into that upon the back whenever the labour becomes instrumental. This practice is certainly open to many objections and attended with no advantages with which we are aware. We have heard that in the Edinburgh Infirmary a regular perforated obstetric chair was employed above half a century ago, and we recollect that some thirty years since it was shown by the late Dr. Hamilton to his midwifery class. In Mr. Michael's work on the Ergot of Rye, it is alleged that in Cornwall even at the present day it is difficult to persuade the female in labour to adopt any other than the standing posture, or a position upon her knees.

Following Dr. Ramsbotham's arrangement we come next to his chapter on the "Irregularities of head presentations," under which he describes, first, vertex presentations with the face behind either groin. This presentation constitutes the third and fourth of the natural positions of the head in the classification of Naegele. We have already shown that the face is very rarely directed to the right groin. On the other hand the presentation of the head with the face looking to the left groin is exceedingly common. If we wish to prove how very little attention has hitherto been paid in this country to the mechanism of natural labour, we might strongly appeal to the errors both in observation and treatment that are to be found in most of our English works, in regard to the presentations in question. Taking one example alone, we find that Dr. Collins in his valuable Report of 16,000 cases of labour in the Lying-in Hospital of Dublin, mentions only twelve cases of presentation of the face anteriorly having been observed. The observations of Naegele, Professor Stoltz of Strasburg, and others, show that when the mechanism of labour is accurately watched, presentations of the face to the left groin alone occur in the proportion of above 20 per cent. of all head cases. Naegele found it in 359 cases out of 1210, or in the ratio of about 1 in every $3\frac{1}{2}$; Dr. Collins detected it only in the ratio of 1 in 1342. In a late valuable paper in the Dublin Medical Journal, Dr. Murphy, formerly assistant in the Dublin hospital, states upon his own observation, that he had discovered this position of the head in the proportion of 1 in 7 out of the 74 cases investigated with a view to this point.

We have already hinted that when the face presents to either groin, it is a general rule, liable to few exceptions, that as the head descends through the cavity of the pelvis, the face comes to be turned round to the nearest sacro-iliac synchondrosis, and the occiput emerges at last under the arch of the pubis, exactly as in those cases in which the face originally presented posteriorly. This spontaneous rectification, as it has been termed, was known to Solayres, but for the knowledge of its great frequency, we are indebted to the zeal and the observation of Naegele. He found the rotation in question take place in 93 out of 96 cases which he carefully watched. In the remaining three instances alone did the face maintain its original position and protrude at the outlet still directed anteriorly. Carus has thought fit to ridicule the mode in which the distinguished Professor of Heidelberg confirmed these curious observations by keeping his fingers in contact with the child's head during the whole course of labour. Too many obstetricians, however, have certified to themselves the truth of Naegele's views, to make ridicule in such a case anything except a proof of the absurd prejudices of him who used it.

A practical fact of the utmost importance has been amply ascertained by Naegele and others, with regard to positions of the head with the face looking to either groin, namely, that such cases are almost universally terminated without any interference on our part, or without much, if any, additional struggle on the part of nature. The change in the position of the head, by which the face turns from the anterior to the posterior part of the pelvis, requires, says Naegele, "no peculiarly favorable circumstances, but these species of labours can be completed by the natural powers under the most usual proportions, in the same time, with the same expense of strength, and without greater difficulty than when the head takes the most common position." We know ourselves from sufficient experience, the fund of truth there is in these remarks. These positions require no peculiar interference on our part; they should not even be considered as morbid; if there is any truth whatever in statistics, we venture to say, from the data we have just adduced, that they daily occur and pass over unobserved in hundreds of instances in which the labour is supposed to be, and no doubt is, perfectly natural; and yet, in many of our text-books, elaborate rules are laid down for their treatment, and some authors doubt the possibility of their completion if they were abandoned to nature alone without the employment of instruments.

Dr. John Clarke published, in the second volume of the Transactions of the Society for the Improvement of Medical Knowledge, an essay on this presentation, in which he recommended a method of manual interference "to be always pursued, when the face is found in the situation above described." By this method, he alleges, he managed to turn the face backwards in 13 out of 14 cases. In 13 out 14 cases nature turns the face backwards without any management of ours. Drs. Burns, Hamilton, and others, have zealously recommended the same unnecessary treatment, and Capuron, Bazignan, and Omoboni, speak of delivery with the face anteriorly as actually impossible without the aid of the forceps or other forms of operative interference.

No accoucheur in this country has probably done more to simplify obstetric practice and disencumber it of operative superfluities than Dr. Blundell. Yet we find this gentleman recommending, in the case under consideration in some instances, rectification of the position by the hand or with the forceps or lever, "when experience and practice are not wanting," or even occasionally by turning "when the softer parts are lax and the pelvis is capacious, and our dexterity from long practice such that we can introduce the hand into the uterus and lay hold of the child's legs and bring it away with facility." (Lee's edition of Dr Blundell's Lectures, p. 65.) Three lines above our last quotation Dr. Blundell ominously recommends us in the treatment of this very same case not to "commit the unpardonable sin of midwifery, the sin I mean of those obstetric reprobates—the meddlesome and pragmatic!"

After speaking of presentations of the face to the groin and to the pubis, and after describing eight varieties of brow presentation (surely a most unnecessary multiplication of them,) Dr. Ramsbotham comes to direct presentations of the face, a class of cases the whole history of which forms one of the best examples to which we could point, of the advantages in obstetric practice of carefully observing how much nature can effect in particular instances, before we attempt to rectify her alleged errors by rude and perilous interference. The history of their treatment in fact is but another illlustration of how very much may be gained by studying obstetric mechanism and the powers of nature.

Many authors of the 17th and first half of the 18th century, seem to have looked upon face cases as so preternatural and difficult that it was generally necessary in the treatment of them, as in the treatment of a hand or shoulder presentation, to have recourse to the operation of turning. Lamotte, Cooper, Smellie, Burton, &c., all appear to have followed this practice, particularly when called in at an early stage of the labour. Giffard, in his posthumous work published in 1734, seems to apologise for finishing two face cases with his extractor or forceps by truly observing, that turning is attended with great difficulty where the " waters are gone off, and the uterus closely envelopes the child." Again Perfect, Baudelocque, and others, while they deem it unnecessary to have recourse to the dangerous operation of turning, still considered active interference necessary, and have, as a common rule, recommended the face to be pushed up and the vertex brought down, contenting themselves thus with a rotation of the head of the infant, and not a rotation of its whole body. Peu speaks of the crotchet—if the face has already descended into the pelvis; and Stein recommends the conjoint use of the fillet and forceps in managing these presentations. Indeed Stein maintained that without some such interference face cases would always be cases of delay and danger. He avers that their spontaneous termination is impossible, except where the fœtus is preternaturally small, or the pelvis preternaturally large. Mesnard endeavoured to prove this by geometrical measurements of the child's head and mother's pelvis, and we regret to add, that one of the most distinguished accoucheurs of the present day, M. Capuron, still upholds the same doctrine, and obstinately opposes the result of his observations with the rule and compass upon the dead and dry head and pelvis, to the result of the direct observations of all his contemporaries upon living nature; thus forgetting or despising one of the great rules of the father of the inductive philosophy—" Non arctandus est mundus ad angustias intellectus, sed expandendus est intellectus ad mundi imaginem recipiendum qualis invenitur."

The sufficiency and success, however, of unassisted nature in face cases was long ago announced to the profession. In 1685, Paul Portal, who in this and in several other matters far outstripped the age in which he lived, taught that in face presentations the child might suffer, and its face be black and swollen, but there is not, he adds, more mystery in this than in a natural or vertex presentation; " I have (he says) delivered several women whose children came with the face foremost, and always without any great difficulty, it being only observed, that in such cases no violence must be used, but nature left to its own course, which done, there is no danger either of mother or child." In 1769 Wallace Johnson in this country, and in 1770 Deleurye in France, both publicly avowed the same opinion in regard to the safety and propriety of leaving to the unaided efforts of nature face presentations; and latterly such an accumulation of statistical proof has been collected upon this question, as must, we think, prove perfectly satisfactory to every unprejudiced mind, and carry conviction to all that prefer the force of simple facts to arguments and dogmatic theories. In 1789 Zeller published 43 cases of face presentations all of which terminated without operative or artificial interference. In 1793 the celebrated Boer declared, that out of 80 face cases, which he had himself observed and noted down, none of the mothers suffered in the slightest degree; three, or at most four, of the children were born dead, and in one case only was artificial assistance (by the forceps) deemed necessary. In 1826, Chevreuil, physician to the Maternité Hospital at Angers, stated that in 18 face cases which he had met with, all terminated naturally. The infants were of the ordinary size; 15 were born alive and 3 were dead, but apparently, he adds, in these cases death had taken place before delivery. In 1836, Dr. Collins of Dublin, in his excellent account of the Cases in the Lying-in Hospital of that city, reports 33 face cases as having occurred during his charge of the institution. All the 33 were delivered without assistance and recovered well. Four of the children were still-born, and one of the four was acepahlous (anencephalous). In one of the four the labour had been allowed to be prolonged thirty-six hours. With the two others it lasted seven or eight hours. In 5 the labour was over within an hour. Some cases of face presentations, Dr. Collins ingenuously adds, I am disposed to think were not noted, delivery having taken place so very speedily.

If it were necessary we might add to the foregoing the statistics in face cases of the Prague hospital, as given by Kilian; or those of Bourg, as reported by Pacoud; and the observations in Copenhagen made by Bang; but it is unnecessary to accumulate further proof, when that proof all tends to the same conclusion. These last three authors conjointly report above 200 face presentations, all showing the great sufficiency of nature in such cases, and the impropriety of rash interference.

In the works before us we find different doctrines of practice inculcated in regard to face cases by Drs. Rigby, Ramsbotham, and Davis. Dr. Rigby gives no direct rules for their management, but his observations all show that he believes face cases to require no particular interference on the part of the practitioner. Dr. Ramsbotham expresses the same opinion, and shows the same repugnance to the employment of any artificial management in ordinary face cases. We have already seen some practitioners advising an attempt at rectifying the position of the head by transforming the face into a cranial presentation. This is a line of practice too often attempted even at the present day. We believe its performance to be impossible after the face has been pushed down into the cavity of the pelvis, when the child and pelvis are both of the natural dimensions, for we believe it physically impossible to turn the fœtal head on its long or occipito-mental axis of five inches through the cavity of the pelvis which is normally only four inches and a half. We quote one or two excellent remarks of Dr. Ramsbotham on this topic:

"It becomes a most important question whether, under a face presentation, any means should be adopted to place the child under a more favorable position. So difficult and almost impossible was the transmission of the head under this presentation at one time thought that it was recommended that the hand should be introduced into the uterus—that the feet should be laid hold of, and that the child should be delivered by turning. This operation, performed under the most favorable circumstances, is always attended with great pain, and frequently with great danger, danger both to the mother and the child: to the mother, from the chance of injury to which her structures (particularly the uterus) are exposed—to the child, in consequence of the pressure which the funis umbilicalis must more or less experience, when the head is passing through the pelvic cavity. All these circumstances, then, being taken into consideration, the practice of changing the position of the child under a face impression by turning is now almost entirely exploded; and we rather leave the case to nature so long as we can safely trust her, than subject the woman and the infant to such dangers. But suppose, on watching the case, we find no advantage gained, no alteration in the position of the head, no advance from hour to hour, what then is to be done? We must here also act upon the same unerring principles elsewhere laid down, wait till symptoms require our interference, and then use that instrument which seems most applicable to the emergency. For it is impossible, by any counter pressure, to make a beneficial change in the situation of the head under a face presentation. We cannot cause the head to turn upon the neck so as to approximate the chin to the chest by pressure." (p. 209.)

We have said that Dr. Rigby has not thought it necessary to offer any rules for the treatment of face cases. Dr. Davis, on the other hand, has laid down rules enough for the management of these cases, but these rules are in our opinion, in various respects, highly objectionable. Indeed if our continental neighbours judge by them of the state of British midwifery, we fear they will consider us as undoubtedly retrograding rather than advancing in this department of the healing art. In order to avoid all risk of misrepresentation we shall here quote his own words:

"When the face is discovered to present at the brim of the pelvis at an early period of a labour, whether before or very soon after the escape of the liquor amnii there can, in the author's opinion, be no doubt as to the preferableness of turning to all other modes of treatment. That operation dexterously performed would, at all events, give the child a good chance of perservation of its life; whilst it would also be the means of rescuing the mother on the very brink of a great impending danger." (p. 659.)

We certainly regret to find such practical doctrines published by an author of Dr. Davis's name and standing, and, above all, by a lecturer on midwifery. The interference he thus teaches is, as we have already seen from the statistical data, useless and unnecessary. We wish this were all we could say against it. We must add our solemn conviction that the practice is not only superfluous but that it is fraught with great danger to both the child and mother. In original footling cases about one in every four is lost from compression upon the cord, and other causes which it is unnecessary at present to enquire into. Dr. Davis advises us to change artificially the face into a footling case, and if so we could not certainly expect to save more than in the natural footling cases. We believe we would save fewer. How strongly then does this degree of danger to the child contrast with the degree of danger to which it was found subjected in Boer's cases, when leaving the case to nature not more than 1 in 20 was lost. In the exercise of our profession we have too often to blame ourselves for errors of omission. Here we think human life is manifestly endangered by an error of commission, if meddlesome and unnecessary interference may bear that name. But the practice affects not the safety and life merely of the child: it affects also the safety and life of the mother. Turning the fœtus in utero is an operation which, as Dr. Churchill calculates it from various data, proves fatal to the mother in about one in every fifteen cases in which it is had recourse to. Are we justified in subjecting any woman to even a fraction of this danger in rectifying or attempting to rectify a presentation, any supposed difficulties connected with which nature every day shows to us she can easily and safely overcome without any assistance on our part?

Let us not be misunderstood. We by no means inculcate that the practitioner remains perfectly apathetic because the presentation is discovered to be a face one. We would rather wish to guard him against the opposite and more dangerous extreme of direct and dangerous interference with the head or body of the child itself. In all such cases we firmly believe, as a general rule, that the *infant* or its position should not be interfered with. We shall not stop to enquire whether the conical-shaped head of the child, when it passes through the pelvis with the face or base of the cone first, is much or in any degree larger than when the vertex or apex of the cone forms its presenting part. But even admitting, what we conceive to be true, that the head when it passes down with the face presenting is slightly larger and more unyielding, and consequently requires a somewhat larger space to pass through than in vertex or cranial cases; still we would strongly repudiate any attempt to manage such cases by intermeddling with the child, while we deem it proper and justifiable to use every simple and safe expedient within our power, which may act upon the *mother* so as to render either the uterine action more decisive, or the parturient passages more dilated, or at least more easily dilateable. For this purpose we avert more carefully all causes that could interfere deleteriously with the functions of the uterus, and at the same time, we also have recourse to bloodletting and other measures calculated to fulfil this indication earlier and more decidedly in face than in cranial presentations, when even there is any tendency to delay: the membranes should be allowed to remain as long entire as possible; the rectum and bladder kept empty; and if, as not unfrequently happens, a fold of the cervix uteri happens to be pushed down before the presenting part, it should be very gently relieved and pushed aside; and the perinæum in this, as in all malpresentations of the head, must be guarded with especial care under the unusual degree of distension to which it is subjected.

Causes of delay and difficulty and danger will happen with presentations of the face, as with presentations of the cranium, and from the very same causes which in cranial cases may render the uterine powers insufficient to complete the expulsion of the child. Under such circumstances our interference or non-interference must be regulated by the same principles as in natural or cranial presentations. We will only add one remark, and we do so in consequence of it being omitted by all the authors whose works we are considering:—If in a laborious face case we use the forceps or the vectis as an extracting instrument, we must hold it ever in our recollection to direct at the outlet the chin and not the forehead under the arch of the pubis. If the chin were turned backwards instead of forwards all our physical efforts at the extraction of the head would be utterly fruitless.

Drs. Davis and Ramsbotham discuss in single pages the nature and symptoms of inefficient uterine action as a cause of lingering labour. The subject is one most highly interesting to the practitioner from the frequency with which it occurs, and important as an object of study from our defective knowledge of its pathology. The various *local* morbid causes of delay and difficulty both on the part of the child or body to be expelled, and of the maternal passages through which that body has to pass, have each and all been made objects of successful investigation by various accoucheurs. We do not recollect one author who has endeavoured, on the other hand, to ascertain and fix the varieties of morbid action and states that, by their presence, may interfere with the organ or organs which are concerned in mechanically expelling the child. And yet we believe, as we have already stated, that, practically speaking, the consideration of this last subject is one of very great moment—perhaps of greater moment than the former.

Dr. Rigby has devoted a very instructive chapter to this topic. It is a chapter which is full of matter calculated to excite the reflection and direct the observations of the obstetric pathologist. We are not certainly prepared to adhere to all the opinions that Dr. Rigby has broached in this part of this work, but we think it requires no stretch of sagacity to predict that it will prove highly useful in rousing the attention of British accoucheurs to the subject, and probably it will be more frequently referred to by after writers than some of the more finished parts of Dr. Rigby's publication.

The mechanical powers by which the child is expelled from the uterus are of two kinds, 1st, the involuntary action of the uterus, assisted, secondly, by the partly voluntary and partly involuntary action of the abdominal muscles and diaphragm. Dr. Rigby, in reference to the morbid conditions of the latter or accessory expulsive powers, states briefly various pathological causes which may more or less impede their action. It would have delighted us if he had entered into this fertile field of enquiry more fully. We have a conviction that the cultivation of it will ere long yield results which will explain the difficulties of many dangerous obstetric cases, and determine more precisely the practice to be pursued in them. The full investigation of the effects of existing cardiac and pulmonary diseases alone upon labour would, we believe, clear up the history and treatment of many at present anomalous cases. Dr. Rigby discusses at considerable length the causes which may retard labour, by inducing a faulty or morbid condition in the involuntary action of the uterus. We wish we had space to extract his views on this matter. For them we earnestly refer our readers to the work itself. We shall give merely some of his opening general observations, to show the spirit in which he treats the subject.

"On the approach of labour, the uterus, which hitherto had been merely performing the office of a receptacle and a means of conveying nourishment to the fœtus, now assumes a totally different character; from being in a nearly passive state, it assumes an entirely opposite condition, namely, of high irritability and powerful action. We might almost suppose, that its connexion with the nervous system was become more close and intimate ; for it is now sensible to the influence of impressions which had before produced no effect upon it. Thus, we see, that affections of the mind, even but of moderate intensity, and to which it was before labour nearly if not quite insensible, are now capable either of rousing its efforts to the utmost violence, or of arresting them in the midst of full activity ; and on the other hand, we see that where its action has been deranged or interrupted, it gives rise to serious affections of the nervous system, or even convulsions. With all this it now displays peculiarities of function which strikingly distinguish it from all other organs of the body; in some cases it appears to annihilate or to absorb, by its all-pervading influence, the functional energies of other organs; and in spite of its increased nervous power and susceptibility to various impressions, it seems to possess the faculty of continuing its efforts uninfluenced by general disease, unimpaired by exhaustion, and for a time almost independent of the life itself of the mother. In convulsions and paralysis, in general fever and inflammation of vital organs, its powers appear to be undiminished; on the contrary, where the patient from whatever cause is rendered incapable of assisting its efforts by the abdominal muscles, the uterus will take upon itself the whole task of expelling the child, which will be born apparently without a single effort on the part of the mother. We also observe that organs, the various conditions and derangements of which have exerted little or no influence upon the uterus in its state of quiescence during pregnancy, now affect it powerfully, and are capable of modifying its action very considerably. The stomach, the intestinal canal, and the skin, are remarkable instances of this, and seldom fail to disturb or prevent the natural efforts of the uterus whenever these organs deviate from a healthy condition. It will be, therefore, of the highest importance to watch their functions narrowly, in order that we may form a correct estimate of their effect upon the uterus." (p. 205.)

Dr. Rigby goes on to show that derangements in the contractile powers of the uterus itself may arise from various morbid states of the organ, both functional and mechanical, as from relative inactivity or debility, from derangement in the digestive organs, from affections of the mind, from age and temperament, from general plethora, or what is probably more frequent, from local uterine congestion, from simple or specific inflammation of the uterine tissues, from irregular or spasmodic contraction in its fibres, from organic disease in its fundus, body or cervix, &c. Each of these divisions receives a separate notice, and their nature, diagnosis, and treatment, are excellently commented on, but for their full discussion we must again refer the reader to the work itself.

In the general management of such cases Dr. Rigby makes one remark which we beg to extract, namely, that "the more carefully such cases are investigated, the less frequently will practitioners require ergot and other oxytoxic medicines." We quote this observation because we believe it strictly true, and because we have seen and heard enough to convince us, that from want of attention to it, the ergot is too often given under circumstances for which it is little or not at all adapted, and where its exhibition is attended with deleterious results both to mother and child. We do not repudiate the use of that drug; we would wish merely, as far as such an observation may serve, to guard against its abuse, and we fear that few medicines are actually more abused in practice. When employed, Drs. Davis and Ramsbotham both recommend the ergot to be given in the form of infusion, and in doses containing half a drachm of the powder, every ten or fifteen minutes. Dr. Davis alleges, that the ordinary mode of exhibiting this drug in America is in an infusion of seven or eight grains of the powder every ten minutes. We have not Prescott's Dissertation at hand to refer to, but assuredly either the dose is accidentally understated, or is in itself far too little to be of any certain avail. Dr. Rigby employs the powder of ergot in cinnamon water (a scruple or two of the former to an ounce and a half of the latter), and adds a few grains of borax. The cinnamon is an old oxytoxic, and long ago the borax was held in high repute by some practitioners upon the continent, for its supposed efficacy in exciting or increasing uterine contractions. Lamotte speaks of its employment, and in our own day its use was again revived by Hufeland, L'Offler, and the late Professor Lobstein of Strasburg. Its alleged efficacy is surely a legitimate subject for direct experiments and observations.

Drs. Ramsbotham and Rigby both treat at considerable length of the various local morbid conditions of the maternal passages and of the fœtus and its appendages, which may impede parturition. As causes of this kind referrible to the ovum, they both describe preternatural thickness and toughness of the membranes; and Dr. Rigby states properly that their premature rupture, as well as their unusual distension with a profuse quantity of liquor amnii, leads occasionally, perhaps more frequently, to the same result. Knots upon the cord are brought forward by Dr. Rigby among his causes of difficult labour from faulty condition of the state of the child. We doubt much the propriety of considering such lesions of the cord under the head in question. They may, as Leveret maintains, occasionally prove hurtful, or even fatal to the child, a result which with Saxtorph, Baudelocque, Gardien, and Rogers, we believe to be extremely rare, but they still more rarely if ever can prove a cause of difficulty in the labour; the knotted cord, as Dr. Rigby himself remarks, generally being of unusual length. This arrangement, as well as that of entering the placenta as a cause of difficult labour, we object to as apt to mislead the young student.

Most modern British accoucheurs have recorded their opinion against the idea of Dr. Denman that shortness of the umbilical cord, either from its original formation or from its being coiled around the neck or limbs of the child, is liable to act as a cause of impediment to the birth of the head at least of the infant. We refer to Dr. Ramsbotham for the usual argument stated against the old opinion, that as the child descends in labour, the fundus and body of the uterus descend with it, until the head is born, so that the same relative distance is maintained during this part of the process of labour, between the two extremities of the cord, as existed during pregnancy. We grant that when very short it may impede the birth of the body, and have seen it do so. We are surprised to see Dr. Rigby on this subject referring to the distinguished Naegele for opinions on it that are common to the general body of English accoucheurs.

As causes of lingering labour on the part of the child itself, we have described in the works under review, strong ossification of the head and its enlargement from natural formation, deformity or disease, accumulations of fluid and tumours of the chest and abdomen, preternatural size of the child, monstrosity; and, lastly, that very rare case which we have described in a former number of this Journal, namely, an anchylosed state of the joints of the fœtus. Dr. Ramsbotham makes some excellent remarks in treating of hydrocephalus as one of the causes in question. It is certainly surprising how little resistance is given by the child's head, even when very considerably distended with fluid; but we are assuredly of Dr. Ramsbotham's opinion, that it is especially dangerous to allow a hydrocephalic head to remain for any considerable time locked in the pelvic cavity. Its compressibility permits it to be moulded into all the irregularities of that cavity, and thus to occasion almost universal pressure on its lining membrane, while the fluidity of its contents adds, on physical principles, to the dangers of these effects. We know one case of this kind, in which a hydrocephalic head produced fatal laceration of the cervix uteri. In another case, where the child presented footling, the spine of the neck, and part of the soft tissues covering it, gave way under the traction employed, and the dropsical head was thus rapidly emptied and allowed to pass.

Accumulations of fluid in the chest or abdomen of the child rarely occasion any impediment to the delivery. In one instance a fœtus, whose abdomen we afterwards found to measure when redistended with fluid nearly twenty-one inches in circumference, could not be delivered until the diaphragm was lacerated. We have met with one case of impediment to the birth of the body of the child after the head was born from great effusion into the thoracic cavities. In reference to this and other causes impeding delivery of the shoulders and thorax, Dr. Rigby very properly advises us to disengage in the first instance the shoulder that lies under the pubic arch.

Instrumental labours form the next subject in Dr. Ramsbotham's arrangement. In some prefatory remarks to this division of his work, he properly animadverts upon reckless operative interference and its miserable effects, and cautions the young practitioner that urgent necessity alone should warrant him in taking an instrument in his hand. In his practice, as Dr. Ramsbotham very truly observes, he will find it much more difficult to determine the time *when* instrumental aid may have become necessary—and, let us add, the *kind* of instrument to be used—than to administer the aid itself. It requires calm and sound judgment to determine the former points; it requires some mechanical dexterity merely to apply the latter.

Dr. Ramsbotham's *first* order of instrumental cases consists of those which are accomplished by instruments perfectly compatible both with the life of the child and the safety and continuity of the mother's structures (including the short and long forceps, the vectis and fillet); his *second* order comprehends those in which either the child's body must be mutilated (embryotomy), or a cutting operation be performed on the mother's person (Cæsarean and Segaultian operations).

In their chapters on the short forceps both Drs. Ramsbotham and Rigby have stated much that is excellent, but little or nothing so novel as to detain us. They each give us a brief history of the forceps, a subject which we have sufficiently discussed on previous opportunities. Dr. Rigby recommends a pair of curved forceps, having the joint invented by Brüninghausen; namely, a combination of the pivot with the common English lock. Dr. Ramsbotham thinks the short forceps should be straight, and says he is in the habit of using Denman's. There is, we believe, as much or more in the hand that uses the instrument than in the instrument itself, but still we could never see how a curved short forceps could be used either with success or safety in those cases (which we have already shown to be so common), where the face presents to either groin. If the instrument is applied to the head in that position, and before the usual turn is made by the face backwards, and if we adhere to Dr. Rigby's rule that the pelvic curvature of the instrument must correspond with the pelvic curvature of the sacrum, it would be impossible, or next to impossible, to give the head its proper rotation as it passed through the pelvis, without the projecting points of the forceps lacerating the passages. We know that in some such cases the head may be brought out with the face still looking anteriorly, but we know well also that in others the greatest physical force will not effect the delivery in this way, when by merely adapting the head, so that it follows the natural mechanism of such presentations, we are speedily enabled to extract the infant. Dr. Davis, perfectly aware of the difficulty we allude to, and particularly of the impossibility of using his own short curved forceps if the head required to be rotated, has proposed to employ two new forms of the instrument with unequal right or left hand blades according as the face presented to the right or left groin. It is almost unnecessary to remark that such complex machinery will never find its way into common practice, even if it possessed any of the varied merit which its author ascribes to it.

Dr. Ramsbotham (p. 297) speaks, in applying the first blade, of first carrying as a director two fingers of the left hand "over the uppermost ear." Dr. Rigby, on the other hand (p. 137), remarks that the position of the head must be determined by the direction of the fontanelles and sutures, and not by feeling for the ear.

"The ear can seldom be reached without causing a good deal of pain, even under the most favorable circumstances. In cases where the head is so impacted as to be incapable of advancing by the natural powers, it cannot surely be justifiable to force up the finger between the head and pelvis to ascertain this point, the more so as the soft parts soon become swollen and more or less inflamed, and therefore little able to bear such rude treatment. No operation requires such an intimate acquaintance with the mechanism of parturition as that for applying the forceps; it is simple and generally perfectly easy when the precise position of the head and its relations to the pelvis are accurately known; on the other hand it is not less injurious and painful to the patient than difficult and unsatisfactory to the practitioner." (p. 138.)

The forceps are always applied in the contrary oblique diameter of the pelvis to that in which the long diameter of the child's head is placed; and Dr. Rigby makes in relation to this circumstance one remark which we think of importance in directing the application of the instrument:

"The trochanter major will guide us as to the precise position of the patient's pelvis, and is especially useful in pointing out the direction of the left oblique diameter, in which the forceps (on account of the first position of the head being in the right-oblique diameter) should be most frequently applied; in this case we pass the upper blade beneath, as it were, the trochanter, and the lower one in the opposite direction." (p. 139.)

Drs. Ramsbotham and Rigby direct attention to the importance of always introducing and extracting the instrument in the line of the pelvic axis. Dr. Davis has given a woodcut very illustrative of the rule. All add strong injunctions with regard to the necessity of care and attention in managing the instrument. The following observations of Dr. Ramsbotham on this point are extremely good:

"Cautiously and tenderly must this iron instrument be used! We must recollect that no sensation can be imparted to the operator's hand of any injury that may be done to the woman; and we must remember that one injudicious thrust, one forcible attempt at introduction, one violent effort in extraction, may bruise, may lacerate, may destroy! Bearing in mind, however, the kind of case in which it is useful and admissible; bearing in mind the principle on which it ought to be employed; recollecting that it is a lever of the first kind; that the metallic blades have no feeling, and cannot communicate to our perceptions a knowledge of any mischief we may inflict, we are not likely to fall into any grave error in its application or its use." (p. 299.)

Dr. Rigby has given few remarks upon the long forceps or their application. Dr. Ramsbotham, however, presents us with an exceedingly valuable chapter on this subject. The long forceps is in British practice an instrument much less generally known, and, as we are inclined to believe, much less generally employed than it ought to be. It is an instrument whose powers should be well known to all. Those who are not prepared to have recourse to its assistance must inevitably, in particular cases in which the head is too high for the short forceps, fall back upon the fearful alternative of mutilating and murdering the infant. Those again who do use it can readily join in the just feeling of pride with which Dr. Ramsbotham speaks of its advantages: "I have," says he, "extracted many children alive by the agency of the long forceps who had been doomed to death by other parties, and who must have been sacrificed to preserve the mother, unless we had possessed this instrument."

The long forceps are especially useful under two sets of circumstances. First, where the child's head is arrested at the brim of the pelvis by any mechanical cause calculated to prevent it passing that obstacle with safety to its own life or to that of its mother, provided its expulsion be left to the unassisted powers of the uterus. The obstacle at the same time must not be so great in degree as to prevent the child's head actually passing if a sufficient additional extractive force be applied. Secondly, the long forceps are, we believe, specially indicated when symptoms or circumstances of complication on the part of the mother arise and show immediate delivery to be necessary, while the head is still at the brim and the membranes have been for some time ruptured.

In construction, the long forceps differ from the short principally in two points, namely, in their greater length and in the divergence of the blades from the shanks, and not from the handles of the instrument. From inattention to this last circumstance, the long forceps of Dr. Haighton cannot, as we have ascertained by experiments upon the dry pelvis, be really employed, for, in consequence of this imperfection in structure, before the head of the child could be well grasped by the blades of the instrument, the parts situated at the outlet of the pelvis are necessarily greatly and injuriously stretched by the diverging shanks. Smellie, Haighton, Hamilton, and Dr. Davis, have recommended long forceps having a double curve, whilst Blundell, Conquest, and Radford use instruments without the pelvic curve; or, in other words, of the same form as the short straight forceps. Dr. Ramsbotham's long forceps have the double curve, and our own experience inclines us to adopt this modification, for we have found that we could fix and use a long forceps possessed of the pelvic curve after a straight pair, from not having such an extensive hold of the head, had repeatedly failed. The long forceps, described and delineated by Dr. Ramsbotham, appear to us to be a good form of the instrument, and we have found it so more than once in practice.

In their mode and place of application, the long forceps differ essentially from the short. The short can be used only after the child's head has completely or nearly completely entered the cavity of the pelvis. Some instruments, indeed, are of such a length, as the forceps of Drs. Orme and Collins, that they could only be applied to the head when it was already pressing upon the perineum. Again, the short forceps are generally if not exclusively fixed over the parietal and temporal bones. On the other hand, the long forceps are applied to the child's head before it has passed the brim, and they must be introduced and adapted, not as the short forceps, in relation to the *head* of the infant, but in relation to the *pelvis* of the mother. In fact, when the pelvis is either of the standard form, or slightly diminished below it, the long forceps are of such a construction that we cannot, with safety to the perineum, apply their blades antero-posteriorly, in relation to the brim ; or, in other words, with one blade behind the symphysis pubis, and another before the promontory of the sacrum. Even in most dry pelves the coccyx prevents the long forceps being applied in this position, provided their extremities be at the same time opened to such an extent as to receive a body the size of the fœtal head. Yet there appears to be no more common error than that the long forceps should be applied in this manner. We have heard it alleged as the proper method by practitioners, who at the same time owned that in those cases in which they examined the hold they had taken of the child's head, they found this marked in such a way by the instrument as to defy their own explanations.

In this country Dr. Davis and Dr. Radford have both shown at length that, in applying the long forceps, we do, as a general rule, seize the child's head more or less antero-posteriorly, and not as with the short forceps, transversely. The simple reason is merely this : from the relative form of the long forceps and pelvis, we can only with safety apply the blades of the former along the *sides* of the brim ; the head presents obliquely, or, when the conjugate diameter of the brim is contracted almost transversely ; and, consequently, either blade must seize the head more or less obliquely on its anterior and posterior aspects. Saxtorph, Stein, and Weidemann speak of applying even the short forceps without any reference to the position of the head, and in a direction regulated by the pelvis alone. We certainly totally disapprove of such a principle as applied to the use of the short forceps, but it is physically impossible to act otherwise when we employ the long to seize the head at or above the brim.

Dr. Ramsbotham states that Deleurye first pointed out in 1779, that in using the long forceps the blades required to be adapted to the forehead and occiput of the child's head. When Smellie, the great reformer and master in English midwifery, advised, still earlier, that occasionally one blade of the long forceps "be introduced *behind* one ear and its fellow *before* the other," he evidently saw a dawning of the truth in regard to this question. The doctrine was opposed by Baudelocque, and Baudelocque's opinion has been too implicitly adopted by Hamilton, Burns, Dewees, &c. We refer to Dr. Ramsbotham's work for a refutation of Baudelocque's views, as well as for the views of others, who, adopting another extreme, think that one blade should be placed over the occiput and the other actually over the face of the child. The very bent position of the child's head upon its breast, and the proportionate large size of the cranium over the face, prevent this last application, and make the anterior blade impinge upon the brow, and not upon the face. "I have employed," says Dr. Ramsbotham, "this instrument on very numerous occasions, and I never, to my recollection, bruised a single feature. In general," he adds, "the point of the instrument has not ascended further than the eyebrow, or (if the head is transversely placed instead of diagonally) than the root of the nose." In a previous part he states, and from what we have ourselves seen we believe correctly, that the blades will be generally found to be applied somewhat diagonally, one reaching to the superciliary ridge or upper part of the orbit, and the other exactly opposite to it on the other side of the occiput.

If our space permitted it would be easy to show, by the most simple mechanical rules, that various authors have promulgated an opinion entirely wrong when they have averred that we have more power with the long than with the short forceps in compressing and injuring the head of the child. The child's head, on the contrary, acts against us with the long end of the lever when the long forceps are applied. They are more dangerous to the child, not from the *degree* of pressure, but from its *direction* being in the antero-posterior axis of the head. The long forceps are assuredly a dangerous instrument to the mother if used injudiciously and incautiously. The same remark applies more or less to every instrument and almost to every drug; but there are some cautions that require to be attended to in the use of the long forceps that do not apply to the short. We shall close our remarks on the instrument by quoting some of these cautions as we find them laid down by Dr. Ramsbotham:

"First. We should not apply the long forceps in a case where great distortion exists. It has already been laid down as a general principle, more than once, that, unless the pelvis possess, in its conjugate diameter, three inches of clear available space, we cannot expect a full-grown well-ossified head to pass entire; and through such a diminished aperture we are not to hope that we shall be able to extract it by the forceps. Burns (Op. cit. p. 440), indeed, fixes the limit of the deformity which would indicate the use of the long forceps at that space. Davis (Operat. Mid. p. 230), from the observations he has given us, would lead us to believe that rather more was generally required than that which Burns specifies; and I am inclined to think that unless the pelvis measures at least three inches and a quarter, we shall generally be foiled in our attempts at delivery; or, at least, be disappointed in our hope of extracting the child living.

"Secondly. In introducing each blade, we must be particularly careful that the point slides within the os uteri, and does not run up between the vagina and the neck of the womb, lest we should bruise or lacerate that organ at its junction with the vagina; and especially lest, in attempting to lock the blades, we should pinch its structure between their extremities and the child's head. This mischance cannot happen with the short forceps, because the os uteri must be entirely dilated before the application, and when the longer pair is used, may be avoided by taking care that the point is constantly kept in contact with the fœtal cranium, guided by two fingers previously inserted.

"Thirdly. We should not employ any strenuous endeavours for effecting delivery, nor work with them for too long a period continuously. The longer the instrument, the greater leverage we possess; and it must be evident that each increase of leverage augments our power: we are not only therefore liable to use too much exertion, but we run the risk of making pressure upon structures less capable of sustaining it uninjured, than when we employ the short forceps. Unless, then, a decided advance be evident after a few minutes' well-directed efforts, we should desist from renewing our attempts; and we must judge of the progress we are making, by examining after each backward and forward movement of the instrument. We must most scrupulously avoid using forcible means.

"Fourthly. We must be guarded in our promises regarding terminating the labour by the means we are about to resort to; because it is impossible that we can measure the head accurately while its base remains above the brim, and it is equally impossible that we should be able to form an opinion of the degree of ossification it has acquired, and of its compressibility. In all these points we may be deceived, although we have made ourselves acquainted with the capacity of the pelvis to the greatest nicety; and while such chances of deception exist, we must be most cautious not to add disappointment to suffering. I have myself, in some instances, been foiled in attempting to extract the head entire through a narrowed aperture, and been obliged, eventually, to have recourse to the perforator, I always feel more satisfied, however, in lessening the head after having made these attempts, because I have good reason to think that nature unassisted would seldom be able to expel a child through a pelvis of such small dimensions as would not admit its passage by the aid of the long forceps; provided in other respects, the case was fitted for the employment of that instrument. I would advise the operator, then, before proceeding to act, not to make a promise of delivery, but merely to state that he is about to do something which will most probably relieve the patient materially, and that, perhaps, he may at once terminate the labour." (p. 337.)

The fillet or lacque is now very generally and very properly discarded from obstetric practice. Dr. Conquest is, as far as we know, the only modern English author who professes to use it. He recommends one made of whalebone, and passed over the chin or occiput of the child, as an occasional substitute for the forceps or lever. (See his Outlines, edit. of 1837, p. 117, and explanation of plate xii.) We doubt if cases in which such an instrument could be thus freely passed, ought to be considered as coming within the range of instrumental labours; and we have no doubt whatever that we have both more efficient and more safe instruments in the forceps and lever, provided artificial assistance were actually required.

Dr. Rigby does not, as far as we observe, speak of either the fillet or vectis. Dr. Ramsbotham has stated in a much more clear and explicit manner than we recollect to have elsewhere met with, the advantages and disadvantages of the vectis or lever as compared with the forceps. We have ourselves no experience of the lever, and hence abstain from offering any remarks upon it. Indeed its employment seems now (and we have made pretty extensive enquiries into this subject) to be confined to the hands of some of the older class of obstetric practitioners. Many of these no doubt use it with great dexterity, but altogether it has always appeared to us a much less useful instrument than the forceps, and, in most respects, one much more difficult and dangerous in its employment.

No surgical operation whatever is, abstractly considered, more revolting to human nature than that of craniotomy or embryulcia: it is at the best a dreadful expedient. In too many instances it implies the direct and deliberate murder of a fellow-being by the hand of the accoucheur. It is one of the few operations, the propriety or non-propriety of which has engaged all the logical subtlety of the metaphysicians. Some of the greatest foreign authorities of the present day doubt if it ever ought to be performed as long as the child is alive. But we have no desire to discuss the question of the morality of the operation. We hold it as a sound and well-established principle in British midwifery that when the lives of both mother and child are in danger, it is lawful and justifiable to sacrifice the life of one in order to save that of the other; and further, in making the selection, the preservation of the life of the parent is ever to be preferred to that of her unborn infant. In the works before us we find nothing new in regard to the indications for craniotomy; and we pass on to a brief consideration of the different modes which the authors follow in the various steps of the operation.

In performing the first part of the operation, namely, the perforation of the head, Drs. Ramsbotham and Davis both advise us to use Smellie's scissors made with a cutting edge to both sides of each blade; Dr. Rigby recommends the perforator of Naegele, the chief peculiarity of which is that, like the analogous instruments invented by Steidele and Von Busch on the continent, and by Drs. Bone and Holmes in our own country, the blades do not cross at the lock, and the operator can, by closing the handles together with one hand, make their cutting points sufficiently diverge, leaving thus free the fingers of the other hand in the vagina to guard and protect the maternal parts from any threatened injury. Both authors properly advise the perforation to be always made in the presenting parietal bone and not at a suture. Dr. Rigby, after perforating, throws a strong stream of water into the cavity of the skull through a long elastic tube, in order to break down and wash out as much as possible of the substance of the brain, and thus allow the cranial bones to collapse more readily and completely.

Dr. Rigby speaks as if it were desirable in all cases to wait a few hours after perforation is performed before we resort to extraction. His remarks cannot necessarily apply to those instances in which the operation is adopted in consequence of some urgent necessity for delivery on the part of the mother; and in cases in which there is only a slight disproportion between the head and pelvis, there is little or no advantage to be gained in delaying the second part of the operation, particularly if the labour-pains still continue. Where the deformity is great, there can, we conceive on the other hand, be no doubt regarding the propriety of the delay, provided there is no direct danger from pressure of the mutilated head upon any of the soft structures of the mother. In these cases, if we operate sufficiently early, the mother's system does not certainly become hourly (as Dr. Ramsbotham expresses it) more depressed. It is a well-ascertained fact, observed in many such cases by many accoucheurs, that in the interval the woman's strength generally recruits, the incipient congestive swelling of the soft parts often disappears, and the commencing putrefaction of the fœtus instead of adding to the difficulties of the extraction usually greatly facilitates it.

In extracting, after perforation, Dr. Ramsbotham uses the common straight crotchet, and well remarks that simple as the instrument is, it is one of the most difficult in midwifery to procure good, as the least variation in the degree of sharpness of its point makes a considerable difference in its value. He states further that the use of the blunt hook may sometimes supersede the employment of the crotchet when the bones are too loose and fragile to afford a purchase to the latter. The craniotomy forceps are in his opinion more dangerous, and less to be trusted to than the crotchet, but the several objections which he makes to these forceps refer to the instrument in its worst form, with a Smellie-joint and immense teeth, and scarcely apply to the simpler and more efficient varieties of it.

In those cases in which there is not great resistance to the passage of the child after its head is reduced, Dr. Rigby advises us to avoid extracting instruments, and to insert the finger only into the perforation of the cranium, and to act with it as a blunt hook. In this way, if assisted by the pains, we may often certainly be enabled to exert a sufficient degree of force to bring the head through the pelvic cavity. In cases, again, where the resistance is greater, and still there is no very unusual degree of deformity, he recommends us to grasp and compress the head with the common curved forceps, and use them as an extractor. "On several occasions," he remarks, "where the craniotomy forceps and crotchet have failed to move the head, the midwifery forceps has been applied, and the delivery easily and quickly accomplished." In fact, occasionally when we fail with the long forceps they may be left on till the skull is perforated, and then used successfully to compress and extract the mutilated head. Dr. Rigby prefers the simple single crotchet when any such instrument requires to be ultimately resorted to.

It would be tedious to enter into the various modifications of crotchets, &c., which Dr. Davis has proposed for extraction; and his opinions on these subjects are already so generally known to the obstetric profession that they need not delay us.

We beg to add only one remark in regard to extraction. Whatever means or whatever instruments are employed to effect it, we have long been of opinion that one great principle should direct their application, namely, they ought to be fixed in such places and used in such ways as will enable us to bring down the head in its most natural and consequently most favorable *position.* We have long been convinced that the great peril not merely of injury but death to the mother from this operation, arises in a great measure from the head being often so altered in its presentation, and hence necessarily increased in its diameters by the mode and place in which the crotchet is fixed, that the cranium is at last torn as it were through the pelvic passages by pure physical force alone, while a little attention to the proper adaptation of the instrument, so that the head may be brought down in a more favorable position will generally be found to lessen immensely both the difficulty and the danger of the operation. We speak here of the more common cases of embryotomy. We are perfectly aware that when the pelvis is much contracted, we are obliged, as has been well pointed out by Dr. Hull, to alter in different ways the presentation of the head, always, however, bringing it into such positions that its diameters are in each case those requiring the least possible space.

Our remarks on the mode of operating by the forceps and crotchet have extended so far as to prevent us entering into the respective indications for the use of the one or other of these instruments. We had wished to have shown statistically that even as regards the *mother* craniotomy has in general proved an infinitely more dangerous operation than the forceps, and we have the greatest doubts of the soundness of the rule that in individual cases the presence of signs of death of the child ought to determine our having recourse to the perforator and crotchet in preference to the forceps. The rule is a dangerous one in two points of view: First, because, as we have just stated, craniotomy seems attended with actually greater risk to the mother; and secondly, because it is exceedingly difficult to make ourselves certain in special instances of the death of the infant. All negative symptoms of its death are very equivocal; even if the cord be felt pulseless still we may not be certain that it is the cord of the child whose head is presenting. Dr. Ramsbotham has properly shown this one source of error, that it may happen to be the cord of another twin infant. The stethoscope in some of these doubtful instances is of the greatest use in determining the presence of *life* in the infant; and in thus encouraging us to persevere as long as possible in other means (as in the use of the long or short forceps) before we have recourse to the final and fatal operation of craniotomy. By its aid in this way we have been induced to use the forceps, and with the happy result of saving the child, when, previously to auscultation, its head had been doomed to perforation by others. We do not however, on the other hand, hold that because the sound of the fœtal heart cannot be heard the indication is of equal value. We do not perform craniotomy in individual cases because the child is dead, but because the mother's life is in danger, and delivery cannot be completed by other means less safe to her. That the employment of the forceps is, as a general rule, safer to the mother herself than the employment of the crotchet is a proposition which Dr. Churchill, of Dublin, and others have proved by ample statistical data. Our German and French neighbours have of late years, in consequence of this and other reasons, been employing the forceps much more and the crotchet much less frequently than we do in Great Britain. We trust yet to see the same reform work its way into English obstetric practice.

We cannot pass from this subject without adverting to a singular mistake into which Dr. Ramsbotham has fallen in speaking of the stethoscope, as "the most satisfactory means of ascertaining the state of fœtal vitality." (p. 361.) He mentions both the "tick" of the fœtal heart and the placental *souffle* as "means that have been resorted to for the purpose of ascertaining whether the child be alive under lingering labour." Now we venture to assert that no one who really knows practically obstetric auscultation ever resorted to the placental *souffle* for the purpose alleged, because it has been long and often proved that this sound may and generally does continue after the death of the child in labour, and until the uterus is contracted and the placenta separated. The presence or absence of the sounds of the fœtal heart is the only proper auscultatory criterion of the presence or absence of fœtal life.

In none of the treatises before us do we find any account of the new continental operation of cephalotripsy, or crushing of the infant's head. It has been practised now with alleged success by the younger Baudelocque, by Champion, Paul Dubois, Halmagrand, Von Busch, and others, and the high names of these men might have entitled it to some passing notice from our authors.

The Sigaultian section in its different modifications is altogether so useless and dangerous an expedient that neither Dr. Rigby nor Dr. Davis treat of it. They both, however, as well as Dr. Ramsbotham, speak at some length of the Cæsarean section. Dr. Rigby gives us some of the statistics of the operation upon the continent and in this country. In the British islands we have only three cases of perfect recovery recorded, viz. in the instances operated on by Mary Dunally, Dr. Barlow, and Mr. Knowles. Six or seven successful operations have been of late years published by different American authors. Dr. Ramsbotham adds an elegant and classical history of the Cæsarean operation in his Appendix. He there makes Shakspeare in his Macbeth declare Macduff as not "one of woman born" for the purpose of enhancing the hero's character by investing his birth with circumstances of an extraordinary nature. This supposition is more ingenious in theory than true in point of fact. The story and almost the very words which Shakspeare uses in the dialogue in which Macduff's alleged Cæsarean birth are brought out were no doubt taken from the Chronicles of Hollinshed, or of Boece whom Hollinshed strictly follows in this subject. Indeed the tale of Boece as set forth in the language of his old translator Bellenden, is certainly far too circumstantial to be historically true, but some of the expressions, as that of "*shorn* from my mother's womb," of the ancient Scottish historian, is little less poetical than the corresponding "untimely *ript*" of the great poet himself.

We have only gone through a small portion of the truly obstetric and practical part of the important volumes before us, and yet the unexpected length to which our remarks have already extended reminds us that, for the present, we must bring our observations to a conclusion. The chapters of Dr. Rigby and Dr. Ramsbotham on Preternatural and Complex Labours contain many interesting and excellent views on some of the forms of morbid parturition included within these two classes. We can only, however, in the meantime refer our obstetric readers to the study of them in the works of the authors themselves, sincerely regretting our inability to lay before them the opinions and practice, in these and other cases, of such distinguished accoucheurs.

In some of our late Numbers, we have had an opportunity of speaking of the various morbid states of the pelvis and soft parts of the mother, that may offer obstacles to the safe and natural delivery of the child, and hence we deem it unnecessary to examine at length those parts of Drs. Ramsbotham and Rigby's works that are devoted to the consideration of this subject. The chapters in both, that relate to it, we can confidently recommend to our readers. No error is more common than that of supposing that exostosis of the pelvis is a frequent disease, and an easily discoverable cause of difficult labour. Naegele has forcibly shown, that most of these supposed exostoses consist merely of a protrusion inwards of the promontory of the sacrum. We are happy to find that a man of Dr. Ramsbotham's extensive experience so far confirms Naegele's views. It is a disease "fortunately," he says, "of very rare occurrence, indeed so infrequent that I have myself never met with an instance." We cannot agree however with the remark that follows, that these exostoses are "generally situated at the back part behind the rectum, and spring from the cavity of the sacrum." In the recorded cases, and in the preparations of them which we have ourselves seen, these exostoses were, in a large proportion of instances, attached to the inner surfaces of one or other of the pelvic joints.

It will be sufficiently apparent, from the whole tenor of our remarks, that we regard the treatises at the head of the present article, particularly those of Drs. Ramsbotham and Rigby, as very valuable additions to English obstetric literature. On certain points, as we have shown, we differ from these authors in their opinions; but, generally speaking, we are happy to find our own principles and practice corroborated by their authority. The freedom with which we may have criticised them on individual topics has arisen more from the position in which we are placed by the very excellence of the works themselves, and the high opinion which we entertain of the judgment and skill of their authors. Dr. Davis's work, though still full of faults, is in its present abridged form in many respects much improved as a compendium of obstetric medicine. Dr. Ramsbotham's treatise we regard as an exceedingly excellent synopsis of practical midwifery. It is rich in minute and sound rules of treatment, and all that the practical accoucheur most needs to know. The very superior style in which the plates are executed reflects the greatest credit on the enterprise of the publisher. Dr. Rigby's work contains so many views comparatively new to the English accoucheur, and brings before us prominently so many of the opinions of the German schools, and is written with such a perfect knowledge, theoretical and practical, of the whole subject, that we entertain little doubt but that it will be soon recognized as an important gift both to the actual practitioner and to the mere student.

---

